# Drug reformulations and repositioning in pharmaceutical industry and its impact on market access: reassessment of nomenclature

**DOI:** 10.3402/jmahp.v1i0.21131

**Published:** 2013-08-06

**Authors:** Susana Murteira, Zied Ghezaiel, Slim Karray, Michel Lamure

**Affiliations:** 1 Equipe Santé, Individu, Société-EA 4128, University of Lyon, Lyon, France; 2 Lundbeck SAS, 37–45, Quai du Président Roosevelt, 92445 Issy-les-Moulineaux, Cedex, Paris, France; 3 Creativ-Ceutical S.A., 215, rue du Faubourg St-Honoré 75008 Paris, France

**Keywords:** repositioning, reformulation, repurposing, combination, lifecycle management, market access, classification, taxonomy

## Abstract

**Background:**

Medicinal products that have been developed and approved for one disease may be the object of additional clinical development in other disease areas or of additional pharmaceutical development for new and different formulations. The newly developed products can be named as repositioned or reformulated products, respectively. Market access of repositioned or reformulated products in Europe and the United States is an interesting object of study as it may provide clarity about which parameters are assessed and considered to bring added value, other than the molecule itself. As such, we aim to evaluate if the added value of repositioned or reformulated medicinal products can be systematically described, quantified, and predicted. As a first step toward investigating the impact of market access on drug research and development trends for repositioned and reformulated products, it is necessary to have consistency in the designations for the case studies evaluated in this project. In an attempt to achieve that consistency, the current study aims to propose harmonized definitions for the repositioning and reformulation strategies and to propose a taxonomy for the medicinal products derived thereof.

**Methods:**

A systematic literature review was conducted to collect information on existing cases of repositioning or reformulation. A search strategy was developed by defining the search objectives, targeted data sources, search keywords, and inclusion/exclusion criteria for the retrieved documents.

**Results:**

A total of 505 publications were retrieved through a search of the main data sources. The screenings and the ad hoc search led to a total of 56 publications to be used for the case study data extraction. In total, 87 repositioning and/or reformulation cases were found described in the literature, 23 of which presented different definitions and/or classifications by different authors.

**Conclusion:**

Given the disparity and inconsistency of terminologies and classifications in the literature, a harmonized nomenclature for drug repositioning, reformulation, and combination cases will allow for a robust analysis of the added value and market access conditions attributed for each strategy and case type as assessed by regulators and payors in Europe and the United States. After evaluation of the existing terminologies and given the absence of clear and consistent definitions for drug reformulation and repositioning in the literature, we propose a global terminology and taxonomy in order to cover all of the previously unclear definitions and classifications for repositioned and reformulated products.

*De novo* drug development is costly ($1.3 billion) and time-consuming (10–17 years) (1–3). In addition, the pharmaceutical industry faces a wide array of challenges, including high rates of drug development attrition during clinical trials, heightened concerns about drug safety, increasing regulatory hurdles, expiring patents, and growing generic competition (4).

As a result of such market forces, pharmaceutical companies are looking for cost-effective and reduced-risk strategies for developing drug products and protecting existing products from competition as well as extending their patent protection time (5). Developing a new formulation or indication for already known drugs can be considered an appealing strategy for drug developers.

‘Reformulation’ is the development of different formulations for the same pharmaceutical drug (6), whereas ‘repositioning’ is the process of finding a new therapeutic use for an already known drug (7). Both are mainstream strategies in drug development (5, 7).

The study of the added value attributed by regulators and payors to potential new development directions for existing drugs is of interest to developers who wish to maximize the potential of their products and sustain their pipelines. As such, identifying opportunities and a rationale for market access of ‘repositioned’ and ‘reformulated’ drugs is a subject of high importance. In addition, market access of repositioned and reformulated products can help to illustrate the parameters that are assessed and considered to bring added value, other than the molecule itself. The regulatory strategies and regulatory pathway trends selected for repurposed drugs might vary considerably and also impact the market access conditions attributted for such products. However, the lack of consensus regarding a harmonized taxonomy for repositioned and reformulated products makes it difficult to identify and review existing cases.

Therefore, we propose a three-part publication with the ultimate goal of understanding the rationale and eventually predicting the impact on market access and conditions attributed to drug reformulation and repositioning in the pharmaceutical industry in Europe and the United States:

Part I: Reassessment of nomenclature

Part II: Regulatory path

Part III: Market access implications

As a first step toward investigating the market access association with drug research and development trends for repositioned and reformulated products, it is necessary to have consistency in the designations for the case studies that are underlying this project. In an attempt to achieve that consistency, the current study aims to propose clear definitions and classifications for repositioning and reformulation strategies and to propose a harmonized taxonomy for the medicinal products derived thereof.

## Methods

A systematic literature review was conducted to collect information on the identified repositioning and reformulation cases. In line with the project's main goal, which is to research reformulation and repositioning cases in order to evaluate the impact on pricing and reimbursement decisions in Europe and the United States, the following objectives were set for the literature research:

1. Collect and describe potential cases of drug repositioning and reformulation mentioned in the literature.
2. Describe and evaluate the circumstances that lead to drug repositioning and reformulation.
3. Describe and evaluate the classification and nomenclature used for drug repositioning and reformulation in the literature.
4. Evaluate the possible implications of existing regulations and guidelines for the development and market approval of drug reformulation/repositioning.
5. Describe the success criteria to be achieved for drug repositioning and reformulation, from a payor perspective.
6. Evaluate the impact of pricing and reimbursement requirements, regulations, and evaluations for the market access of repositioned drugs and reformulated drugs, and if these can be forecasted.
7. Evaluate the potential lost benefit for patients and the society as a whole in cases where pricing or reimbursement cannot be achieved.

The present study focuses on objectives 1, 2, and 3 in order to evaluate the consistency of nomenclature of drug reformulation and repositioning cases.

The literature review was conducted from May 14, 2012, to May 25, 2012. The search was conducted by screening the Embase online database. Given the wide scope of this project, a review of the literature listed in Google Scholar and Google, as well as some proprietary data sources, was also performed. This *ad hoc* search facilitated the retrieval of publications covering some of the topics of interest. In line with the objectives of the project, a list of keywords and a search algorithm were established (Table I).

**Table I:** Embase search keywords and search algorithm

Search strategy number	Query	Number of results	Date
1	‘drug repurposing’ OR ‘drug reformulation’ OR ‘drug repositioning’/exp OR ‘drug repositioning’ OR (reformulat* OR reposition* OR ‘line extension’ OR repurpos* OR ‘new indication’ OR ‘new formulation’ AND (‘drug’ OR ‘drug’/exp OR drug)) AND [embase]/lim	4209	14, 15 May 2012
2	‘pharmacoeconomics’/exp OR ‘pharmacoeconomics’ OR ‘health care cost’/exp OR ‘health care cost’ AND (‘cost’ OR ‘cost’/exp OR cost) OR price OR pricing OR ‘reimbursement’ OR ‘reimbursement’/exp OR reimbursement AND [embase]/lim	194108	16, 17 May 2012
3	‘regulation’ OR ‘regulation’/exp OR regulation OR regulatory OR ‘authority’ OR ‘authority’/exp OR authority OR authorization OR ‘formulary’ OR ‘formulary’/exp OR formulary OR listing AND [embase]/lim	1688208	18, 21, 22 May 2012
1 and (2 or 3)	‘ding repurposing’ OR ‘drug reformulation’ OR ‘drug repositioning’/exp OR ‘drug repositioning’ OR (reformulat* OR reposition* OR ‘line extension’ OR repurpos* OR ‘new indication’ OR ‘new formulation’ AND (‘drug’ OR ‘drug’/exp OR drug)) AND [embase]/lim AND (‘pharmacoeconomics’/exp OR ‘pharmacoeconomics’ OR ‘health care cost’/exp OR ‘health care cost’ AND (‘cost’ OR ‘cost’/exp OR cost) OR price OR pricing OR ‘reimbursement’ OR ‘reimbursement’/exp OR reimbursement AND [embase]/lim OR (‘regulation’ OR ‘regulation’/exp OR regulation OR regulatory OR ‘authority’ OR ‘authority’/exp OR authority OR authorization OR ‘formulary’ OR ‘formulary’/exp OR formulary OR listing AND [embase]/lim)	505	25 May 2012

A list of relevant keywords were tested and then used in an attempt to address the search objectives. The developed search algorithm for the online Embase database was the combination of three keyword-lists.

Only those documents that addressed at least one of the research objectives and met specified inclusion and exclusion criteria were included for analysis (Table II).

**Table II:** Inclusion and exclusion criteria for the analysis

	Inclusion Criteria	Exclusion Criteria
Content	Meets any of the research objectives	New candidates for repositioning or reformulations is drugs (still in development or still pending for approval)
Geographical Scope	EU+USA	Out of EU or USA
Publication language	English or French	Other than English or French

The content, geographical scope, and language were our main criteria for selection or rejection of publications during the screening process.

All published or not formally published documents (institutional reports, technical reports, etc.) were considered for the search. For the initial selection, it was deliberately decided not to have a time limit regarding the time of publication. Any literature that mentioned only potential candidates for drug repositioning or reformulation were excluded given the impossibility to assess the impact on market access for these products.

All the sourced references were imported to a Data Manager file. The list of titles and abstracts were screened (1st selection process) and once the full publications were collected, according to the process previously described, they were thoroughly screened (2nd selection process).

## Results

### Overview of literature search results

**Fig. 1:**
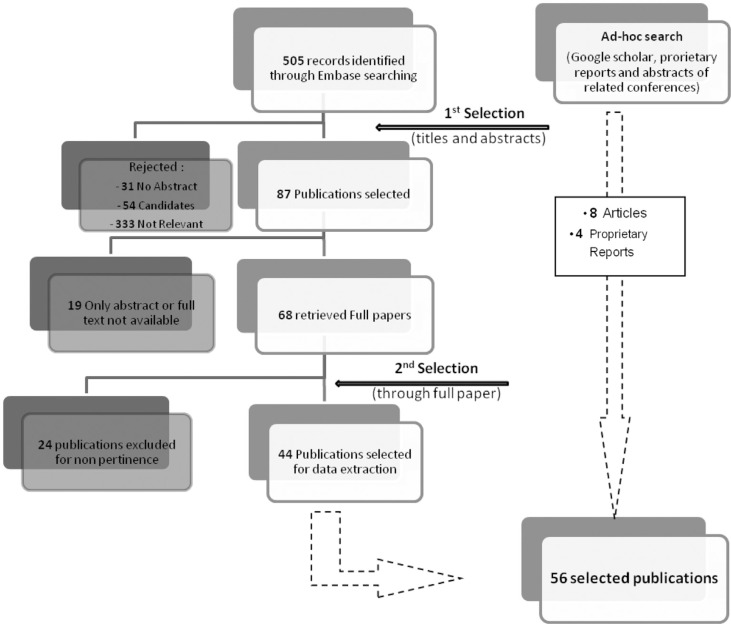
Flow diagram of reviewed publications. The diagram shows the number of selected publications at the different search phases, including the Embase search results, and the additional articles retrieved using the ad hoc search.

The search in the Embase database sourced 505 publications. After screening for duplicates and analyzing titles and abstracts, 87 publications were selected. The reasons for rejection were recorded (Fig. 1). From the selected publications, 19 were not available in full text format, leading to 68 full articles being retrieved. The second selection process excluded 24 more publications, leading to selection of 44 full articles from Embase (Supplemental material 1). The *ad hoc* search allowed for the inclusion of eight more pertinent articles and four proprietary reports, which resulted in a total of 56 publications for data extraction.

### Overview of selected publications

**Fig. 2:**
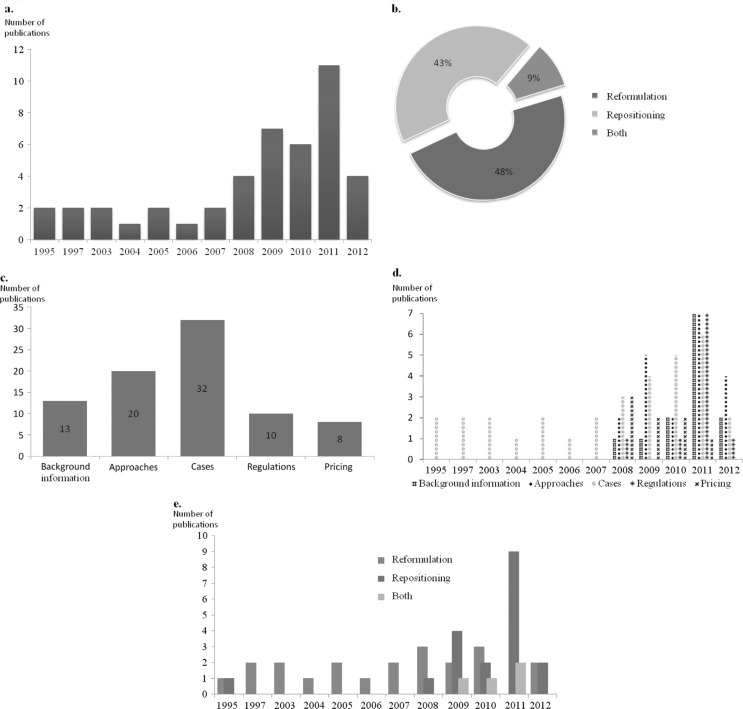
Overview of selected publications. (A) Distribution of publications by year of publication. (B) Distribution of publications by scope: The selected publications are distributed depending on their scope as attributed by us during the data extraction phase. The scope can be drug reformulation, drug repositioning, or both. (C) Distribution of publications by reason of selection; during the screening phases of the search, reasons for selection were recorded and we attributed five categories covering the overall search objectives: Background information, including definitions and general information; Approaches, any description of strategies for reformulations or repositioning; Cases, all case studies or examples of repositioned or reformulated products; Regulations, any regulatory implication; Pricing, pricing and market access of repositioned and reformulated products. (D) Distribution of publications by reason for selection and year of publication. (E). Distribution of publications by year of publication and scope (reformulation, repositioning, or both).

The selected documents were published between 1995 and 2012. Fig. 2A shows that more than 50% of the articles were published during the years 2009, 2010, and 2011.

As shown in Fig. 2B, the retrieved articles were almost equally distributed between cases of reformulation and repositioning. A total of 32 of the selected publications focused on the description of cases of reformulation and/or repositioning (Fig. 2C). In contrast with the publications describing in detail the cases for reformulations and repositioning, the remaining publications concerning regulatory process, background information, type of approaches or pricing, were published after 2008 inclusive (Figure 2D). It is noteworthy that publications concerning repositioning were mainly published during the last 5 years (Fig. 2E).

### Description of cases found in the literature

Our results included 125 cases of reformulation and repositioning. By using basic definitions for reformulation, ‘a new formulation of existing product’, and for repositioning, ‘a new indication for a known drug’, the number of cases was reduced to 87 after eliminating duplicates. In particular, some cases of repositioning such as sildenafil, thalidomide, and imatinib were frequently found. Table III contains detailed information about the reformulation and repositioning for the examples cited below.

**Table III:** Examples of cases of reformulation/repositioning found in the literature

Drug (s)	Original indication	New indication	Original formulation	New formulation	Ref.
Amiodarone	Initiation of treatment and prophylaxis of frequently recurring ventricular fibrillation and hemodynamically unstable ventricular tachycardia in patients refractory to other therapy.	–	Cordarone^®^ (oral formulation)	Cordarone IV^®^ (IV formulation)	(11)
Azelastine hcl	Use in patients with seasonal allergic rhinitis (SAR) and non-allergic vasomotor rhinitis (VMR)	–	Azelastine (Astelin^®^ 0.1%) Bitter taste (Side effect) Saline (Excipient)	Astepro^®^ 0.1% sucralose and sorbitol (Excipient)	(10)
Citalopram Escitalopram	Major depressive disorder (MDD)	–	Citalopram (Cipramil^®^ or Celexa^®^) Racemic mixture	Escitalopram (Cipralex^®^ or Lexapro^®^) Single enantiomer version	(9, 12)
Cyclosporine A	Prophylaxis of graft rejection	–	Oral cyclosporine (Sandimmune SGC^®^)	Microemulsion oral formulation (Neoral^®^)	(13)
Efavirenz, emtricitabine and tenofovir diprovoxil fumarate (Atripla^®^)	HIV	–	Efavirenz, emtricitabine (available individually or in combination with tenofovir diprovoxil fumarate) and tenofovir diprovoxil fumarate (available individually as or in combination with emtricitabine)	(efavirenz/emtricitabine/tenofovir) Atripla^®^	(14)
Mesalazine	Induction of clinical and endoscopic remission in patients with mild-to-moderate, active ulcerative colitis (UC) and for the maintenance of remission	–	Oral mesalazine (Asacol^®^) 400/800-mg tablets 2–3 times daily	MMX Mesalazine (Mezavant XL^®^) 1,200-ms tablets once daily using multi-matrix release (MMX) technology	(15)
Omeprazole Esomeprazole	Gastric, anti-secretory	–	Omeprazole (Losec^®^ or Prilosec^®^)	Esomeprazole Nexium^®^ Single enantiomer version	(9)
Paroxetine	Major depressive disorder (MDD)	–	Paxil^®^ or Seroxat^®^ immediate release formulation	Paxil CR^®^ (extended- or controlled release formulations)	(9, 12)
Valproate	Epilepsy	–	Chrono^®^ (Tablets) [not adapted to children]	Oral solution or syrup (immediate-release formulations, twice to three times daily) Chromosphere^®^ (microspheres powder) modified release formulation	(16)
Venlafaxine	Major depressive disorder (MDD)	–	Effexor^®^ immediate release formulation	Effexor XR^®^ Extended Release formulation	(9, 12)
Crizotinib	Anaplastic large-cell lymphoma	Non-small-cell lung cancer (NSCLC)	–	–	(1)
Duloxetine	Major depressive disorder (MDD)	Stress urinary incontinence (SUI) Yentreve^®^ in Europe	–	–	(1, 5)
		Fibromyalgia;			(1, 5)
		Chronic Musculoskeletal pain			(1, 5)
		Maintenance treatment of MDD;			(5)
		General Anxiety Disorder (GAD)			(5)
		Maintenance treatment of GAD;			(5)
Everolimus	Immunosuppression	Pancreatic neuroendocrine tumours			(1)
Finasteride	Benign Prostatic Hyperplasia (BPH)	Male Pattern Baldness (MPB)	Proscar^®^ 5 mg tabs	Propecia^®^ 1 mg tabs	(17)
Desloratadine	Allergies	–	Loratadine (Lorastine^®^ or Claritin^®^)	Desloratadine (Clarinex^®^, NeoClarityn^®^ or Aerius^®^) Active metabolite of loratadine	(9)
Bromocriptine (Parlodel^®^ -Cycloset^®^)	Parkinson's disease, hyperprolactinaemia and galactorrhoea	Diabetes mellitus (T2DM)	Standard release (Parlodel^®^)	Quick release (qr) (Cycloset^®^)	(7)
Hydralazine and isosorbide dinitrate	Hypertension vasodilator	Heart failure	25, 50 mg tab (hypertension) 20 mg tab (vasodilator)	37.5/20 mg tab	(5)
Minoxidil	Hypertension	Male pattern baldness (MPB)	Tablet	Topical use	(1, 7, 18)
Paclitaxel	Cancer chemotherapeutic agent	Prevention of restenosis of coronary stents	–	A unique form of delivery (stent elution)	(5)
Imatinib	Chronic Myeloid Leukemia (CML)	Gastrointestinal stromal tumor (GIST)	–	–	(1, 3, 17, 18)
		Ischaemic stroke	–	–	(1, 3, 17, 18)
		Rheumatoid arthritis	–	–	(1, 3, 17, 18)
		Psoriasis	–	–	(1, 3, 17, 18)
		Crohn's disease	–	–	(1, 3, 17, 18)
		Type I diabetes	–	–	(1, 3, 17, 18)
		Spondyloarthritis	–	–	(1, 3, 17, 18)
Paclitaxel	Cancer chemotherapeutic agent	Treatment of metastatic breast cancer	–	–	(18)
Sildenafil	Angina (failed clinical trials)	Erectile dysfunction	–	–	(1, 4, 7, 17–21)
	Erectile dysfunction	Pulmonary arterial hypertension	50–100 mg tabs	20 mg tabs	(1, 19)
Sunitinib	GIST, renal cell carcinoma	Pancreatic neuroendocrine tumors	–	–	(1)
Thalidomide	Prescribed to pregnant women for preventing morning sickness (withdrawn)	Erythema Nodosum Leprosum (ENL)	–	–	(1, 5, 7)
		Multiple myeloma	–	–	(1, 5, 7)
Trastuzumab	HER2-positive breast cancer	HER2-positive metastatic gastric cancer	–	–	(1)

Repositioning and reformulation cases that were cited in the results section are presented in this table with details including the indication and formulation of the original product and the new indication and/or formulation.

### Description of cases regardless the nature of change

The aforementioned 87 cases were analyzed and classified based on different parameters as detailed below. Accordingly, Table IV illustrates the diversity of the cases through some of the most popular cases of repositioning and reformulation, carefully selected to highlight the different possible situations.

- **Timing of launch of the new product**
  Repositioning or reformulation can be done with drugs that were never commercialized for their originally developed goals. This was the case with sildenafil for its first repositioning from angina to erectile dysfunction. Several cases were found for marketed products, including specific situations of discontinuations. A notorious example of that situation is that of thalidomide, which was repositioned twice after being tragically withdrawn from the market (1, 5, 7).
- **Availability of generics of the original product when the change occurred**
  In the case of bromocriptine (7, 8), generics of Parlodel^®^ were already available when the new product was launched. It is common that line-extension drugs are introduced prior to the introduction of generics (9). For example, Azelastine (Astelin^®^) was reformulated 1.5 years prior to patent expiry (10).
- **Company developing the new product versus the original company**
  A product can be repositioned or reformulated by the company that owns the original product or by a different company. For example, paclitaxel originally marketed by Bristol-Myers Squibb was repositioned by Boston Scientific (18).
- **Use of same or different brand name as the original product**
  Regarding reformulations, in most cases the product keeps the same brand name with a minor change; for example, Effexor XR^®^ and Paxil CR^®^ are the new formulations of Effexor^®^ (venlafaxine) and Paxil^®^ (paroxetine), respectively (9, 12, 22).

**Table IV:** Distribution of some cases according to different parameters

		Type	Marketing status of original product at launching time new product			Genericization status at launching time new product			Company marketing the new product		Brand name of new product	
					
	Reformulation	Repositioning	pre-launch	post-launch	With-drawn	No Generics eminent	Generics eminent	Generics available	Some as original	Different from original	Same as original	Different from original
Sildenafil (Viagra^®^)		X	X			X			X		–	–
Sildenafil (Revatio^®^)	X	X		X		X			X			X
Thalidomide (for ENL)		X			X	X			X			X
Thalidomide (for Multiple myeloma)		X		X		X			X		X	
Finasteride	X	X		X					X			X
Azelastine	X			X			X		X			X
Duloxetine		X		X		X			X		X	
Bromocriptine	X	X		X				X				X

An illustrative list of repositioning and formulation cases is analyzed according to the parameters explained in the section ‘Description of cases regardless the nature of change’.

### Overview of repositioning cases

The events and processes leading to the repositioning of a product are diverse. Repositioned products are classified based on three main criteria.

- **Approach leading to the discovery of the new indication**
  Serendipitous discovery accounts for some of the most prominent cases of repositioning, such as that of sildenafil (for erectile dysfunction) and minoxidil (1, 3, 5, 7, 18).
  In contrast, a rational approach relies on the understanding of the disease physiopathology and/or the drug mechanism. Imatinib is an illustrative example: the understanding of the role of KIT tyrosine kinase was the trigger for initiating the preclinical studies for gastrointestinal stromal tumors (1, 3, 17, 18, 23).
- **Pharmacological target compared to the original product**
  Several drugs, including duloxetine, sunitinib, and everolimus, were found to be effective in another disease via the same pathway or protein interaction. This is what some authors refer to as ‘on-target repositioning’ (1, 5).
  ‘Off-target repositioning’ is defined as a new therapeutic use based on a newly discovered (or previously unexplored) additional pharmacological mechanism for a known drug (24). This was the case for imatinib, which was found to address new targets in addition to the previously known BCR-ABL fusion protein inhibitor (1, 3, 17, 18).
- **Therapeutic area compared to the original product**
  In many cases, the new and original indications are within the same therapeutic area [trastuzumab, sunitinib (1), and paclitaxel (18)]. A drug can be found to have a different pharmacological target but still within the same therapeutic area. An example of this situation is crizotinib, which is still being tested for its original intended indication as a treatment for anaplastic large-cell lymphoma via its known target, the MET kinase, and was repositioned for the treatment of non-small-cell lung cancer via a new target, the EML4-ALK oncogene (1).
  The same drug product can be found effective in a different therapeutic area. There are many examples of on-target repositioning that fit in this category, including that of finasteride (5, 17, 18).

### Overview of reformulation cases

Cases of reformulation are particularly frequent in the realm of psychiatry because non-adherence to treatment is a key issue in the treatment of mental illnesses (9, 12, 25).

- **Modified release formulations**
  Release modifications represent more than half of the cases of reformulations found. This approach often comprises the use of sophisticated formulation technologies and/or changes in excipients to modify the release rate of the active substance. For example, the use of multi-matrix release (MMX) technology for mesalazine tablets (Mezavant XL^®^) (15).
- **Change in pharmaceutical form and/or change in administration route**
  Changes in the pharmaceutical form can be varied and can include no change in the administration route, such as the microemulsion form of cyclosporine A and the syrup form of valproic acid, both for oral use (13, 16). A change in the administration route can be included too, such as amiodarone's new IV formulation versus the original oral tablets (11).
- **Excipients change without impact on pharmacokinetic parameters**
  In some cases, the reformulation concerns the modification of particular ingredients without modification of the product's pharmacokinetic profile, administration route, or the pharmaceutical form. This was the case of azelastine (Astelin^®^) when reformulated to Astepro^®^ (10).
- **Change in the structure of the active substance**
  In contrast to all the reformulation cases cited above, the structure of the active pharmaceutical ingredient (API) can be modified to form a new formulation. This modification ranges from a ‘simple’ chiral switch, such as escitalopram and esomeprazole (9, 12, 26), to a more significant chemical change via prodrugs or active metabolite strategies, such as desloratadine (Aerius^®^) (9).
  The change of specific ingredient and structural change of the active compound may involve the change of chemical, physical, or clinical parameters as illustrated in Table V. Depending on the reformulation approach used, some changes to the pharmacokinetic parameters can be involved (see Table VI).

**Table V:** Overview of change of chemical or physical or clinical properties

			Parameters changed by the reformulation		
			
Active substance	Brand name	Reformulation Type	Chemical	Physical	Clinical
Mesalazine	(Mezavant XL^®^)	Modified release		X	X
Cyclosporine A	(Neoral^®^)	Change in pharmaceutical form		X	X
Azelastine	(Astepro^®^)	Change of excipient		X	X
Vinorelbine	(Navelbine^®^ Oral)	Change in administration route		X	X
Escitalopram	(Cipralex^®^ or Lexapro^®^)	Product simplification		X	X
Desloratadine	(Clarinex^®^, NeoClarityn^®^ or Aerius^®^)	Active metabolite	X	X	X

An illustrative list of cases from different reformulation types is presented according to the parameters modified by the reformulation (chemical, physical, and clinical).

**Table VI:** Overview of the changes according to the targeted pharmacokinetic parameters

		Pharmacokinetic parameters affected by the reformulation			
		
	Reformulation type	Absorption	Distribution	Metabolism	Elimination
Mesalazine (Mezavant XL^®^)	Modified release	X	–	–	–
Cyclosporine A (Neoral^®^)	Change in pharmaceutical form	X	–	–	–
Azelastine (Astepro^®^ 0.1%)	Change of excipient	–	–	–	–
Vinorelbine (Navelbine^®^ Oral)	Change in administration route	X	–	–	–
Escitalopram (Cipralex^®^ or Lexapro^®^)	Product simplification	X	NA	–	X
Desloratadine (Clarinex^®^, NeoClarityn^®^ or Aerius^®^)	Active metabolite	X	X	X	X

An illustrative list of cases from different reformulation types is presented according to the pharmacokinetic parameters affected by the reformulation (absorption, distribution, metabolism, and elimination).

### Overview of drug combination cases

Drug combination is considered by many authors as a re-launch strategy and is a recurring theme in repositioning of drugs (24, 27). The combination can be approved for use in a different (such as Bidil^®^) (5) or in the same indication granted for the individual components (such as Atripla^®^) (14).

## Discussion

### Evaluation of the terminology and classification found in the literature

Many definitions were available in the literature for drug repositioning and reformulation. There was an unclear definition and limitation on what is to be considered ‘true’ repositioning or reformulation, if these can include or exclude ‘simple’ line extensions and indicate how to define and denominate the cases when the drug is simultaneously developed through a repositioning and a reformulation process. Additionally, it wasn’t clear in the literature in which classification category can drug combinations be included. This was also the situation for the cases with structural changes of the active ingredient such as chiral switch, prodrugs or use of active metabolites.

This lack of consistency was also observed in the classification criteria of the cases. Some authors based their classification on the change of pharmacokinetic properties, while others used a classification based on the timing of the original product's lifecycle. Furthermore, other authors based their classification on the differences in the approach used for discovery of indication or on the commercial success of the repositioned product.

Our research findings confirmed the non-availability of a complete and homogeneous definition for both drug repositioning and reformulation. Thus, different authors have been developing and using different, and sometimes, even contradictory definitions (5, 7, 17, 24, 28, 29). A summary of the non-consistencies found in terms of definition and classification is shown in Table VII.

**Table VII:** Non-consistency in the literature regarding repositioning and reformulations illustrated by cases found in our results

Drug (s)	Original indication	New indication	Original formulation	New formulation	Type (Ref./Rep./Ref.-Rep.)^a^	Description of the reformulation or repositioning	Definition or classification	Reference
Azelastine hcl	Use in patients with seasonal allergic rhinitis (SAR) and non-allergic vasomotor rhinitis (VMR)	–	Astepro^®^ 0.1% twice daily	Astepro^®^ 0.15% once daily	Ref.	Higher dose, different dosing regimen	Dose change being considered as a reformulation	(10)
			Azelastine (Astelin^®^ 0.1%) Bitter taste (Side effect) Saline (Excipient)	Astepro^®^ 0.1% sucralose and sorbitol (Excipient)	Ref.	Change of sweetener agent	Change of ingredient considered as reformulation	(10)
Dabigatran etexilate (Pradaxa^®^)	Thromboprophylaxis in adults following a hip or knee joint replacement	Prevention of stroke and systemic embolism in patients with non-valvular atrial fibrillation (AF)	–	–	Rep.	New indication, Similar therapeutic area	New use in similar indication considered as repositioning	(30)
Bevacizumab	Treatment of metastatic colorectal cancer	Multiple cancers (approved) Gastric Cancer (failed)	–	–	Rep.	Repositioning within similar therapeutic area (oncology)	New use in similar indication considered as repositioning	(1)
		Treatment of choroidal neovascularization in age-related macular degeneration (Off label)	–	–	Rep.	Repositioned but Off label use for the new indication (Not approved)	Approval status for the new use	(18)
Bromocriptine (Parlodel^®^-Cycloset^®^)	Parkinson's disease, hyperprolactinaemia and galactorrhoea	Diabetes mellitus (T2DM)	Standard release (Parlodel^®^)	Quick release (QR) (Cycloset^®^)	Rep.	New indication	Repositioning case, regardless the formulation change by release modification	(7)
					Ref.-Rep.	New formulation and indications for existing product	Modified release formulation for a new use considered as repositioning and reformulation	(8)
Bupropion hcl	Major depressive disorder (MDD)	Major depressive disorder (MDD)	Wellbutrin^®^	Wellbutrin^®^ (once a day dosing)	Rep.	New formulation (modified release)	Reformulation as a repositioning strategy	(5)
		Smoking cessation	Wellbutrin^®^, Wellbutrin SR^®^	Wellbutrin SR^®^ 150 mg	Rep.	New indication	Repositioning case, regardless the change of pharmaceutical form and dosage	(7)
					Ref.-Rep.	Change of dose Modified release with a new indication	Change of dose with release modification for a new use	(5)
Buproprion + naltrexone	Opioid addiction	Obesity	–	–	Rep.	Combination for synergistic effects in a new indication	Repositioning via combination for a new indication	(1)
Crizotinib	Anaplastic large-cell lymphoma	Non-small-cell lung cancer (NSCLC)	–	–	Rep.	Repositioning within similar therapeutic area (oncology)	New use in similar indication considered as repositioning	(1)
Desloratadine	Allergies	–	Loratadine (Lorastine^®^ or Claritin^®^)	Desloratadine (Clarinex^®^, NeoClarityn^®^ or Aerius^®^) Active metabolite of loratadine	Ref.	Active metabolite	Active metabolite considered as a reformulation strategy	(9)
Doxep in (Sinequan^®^-Silenor^®^)	Major depressive disorder(MDD) and/or anxiety	Insomnia	10, 25, 50, 75, 100 mg capsules	Low dosage 3 and 6 mg tablets	Ref.-Rep.	New formulation (lower dose) and indications for existing product	Dose change being considered as a reformulation	(5)
Duloxetine	Major depressive disorder (MDD)	Stress urinary incontinence (SUI), Fibromyalgia, Chronic Musculoskeletal pain	–	–	Rep.	Repositioning in different therapeutic area	New use in a different therapeutic area considered as repositioning	(1, 5)
		Maintenance treatment of MDD; general anxiety disorder (GAD); maintenance treatment of GAD	–	–	Rep.	Repositioning in similar therapeutic area (psychiatry)	New use in similar indication considered as repositioning	(5)
Efavirenz, emtricitabine and tenofovir diprovoxil fumarate (Atripla^®^)	HIV	–	Efavirenz, emtricitabine (available individually or in combination with tenofovir diprovoxil fumarate) and tenofovir diprovoxil fumarate (available individually as or in combination with emtricitabine)	Efavirenz/emtricitabine/tenofovir	Ref.	Combination for the same indications as the mixed compounds	Fixed Dose Combination considered as reformulation	(14)
Enoxaparin	Prevention of venous thromboembolism (VTE) after total hip replacement	Prevention of deep venous thrombosis (DVT) following knee replacement surgery	–	–	Rep.	New indication in same therapeutic area	New use in similar indication considered as repositioning	(31)
Etanercept (Enbrel^®^)	Indicated to reduce the signs and symptoms, inhibit the progression of structural damage of active arthritis (Nov 1998)	- Improve physical function in patients with psoriatic arthritis; - Moderately to severely active polyarticular-course juvenile rheumatoid arthritis - Active ankylosing spondylitis - Moderate-to-severe plaque psoriasis	–	–	Rep.	Repositioning within relatively similar therapeutic area	New use in similar indication considered as repositioning	(3)
		Sciatica	–	Epidural delivery	Ref.-Rep.	New administration route for the new indication	New delivery route for a new use considered as repositioning and reformulation	(3)
Finasteride	Benign Prostatic Hyperplasia (BPH)	Male Pattern Baldness (MPB)	Proscar^®^ 5 mg tabs	Propecia^®^ 1 mg tabs	Rep.	New indication for existing product	Repositioning (not associated with reformulation)	(17)
					Ref.-Rep.	New formulation (lower dose) and indications for existing product	Lower dose considered as a reformulation	(5, 18)
Hydralazine & Isosorbide dinitrate	Hypertension/vasodilator	Heart failure	25, 50 mg tab (Hypertension)/20 mg tab (vasodilator)	37.5/20 mg tab	Ref.-Rep.	Repositioning and reformulation	Combination for a different therapeutic area considered as repositioning and reformulation	(5)
Naltrexone (Revia^®^-Vivitrol^®^)	Opioid addiction and alcohol dependence	Prevent opioid dependence relapse and alcohol dependence	50 mg tab	Once per month extended release injectable	Rep.	New formulation of an existing product	Reformulation considered as a type of repositioning	(5)
Paclitaxel	Cancer chemotherapeutic agent	Prevention of restenosis of coronary stents	–	A unique form of delivery (stent elution)	Rep.	New formulation (new form) and indications for existing product	Repositioning case, regardless the change of form	(18)
		Treatment of metastatic breast cancer	–	–	Rep.	Repositioning within similar therapeutic area (oncology)	New use in similar indication considered as repositioning	(18)
Paliperidone	Schizophrenia	–	Risperidone (Risperdal^®^)	Paliperidone (Invega^®^) contains risperidone's known active metabolite	Ref.	Active metabolite	Active metabolite as a reformulation strategy	(9)
Sildenafil	Angina	Erectile dysfunction	–	–	Rep.	Side effect during clinical trial, compound that failed in its lead indication	New use for a compound that failed in clinical trials, considered as repositioning	(1, 4, 7, 19–21)
	Erectile dysfunction	Pulmonary arterial hypertension	50–100 mg tabs	20 mg tabs	Rep.	New indication, On target, Informed insight	Repositioning case, regardless the change of dose	(1, 19)
					Ref.-Rep.	New formulation and indications for existing product	Dose change being considered as a reformulation	(5)
Sunitinib	GIST, renal cell carcinoma	Pancreatic neuroendocrine tumors Multiple cancers	–	–	Rep.	Similar therapeutic area (oncology)	New use in similar indication considered as repositioning	(1)
Trastuzumab	HER2-positive breast cancer	HER2-positive metastatic gastric cancer	–	–	Rep.	Similar therapeutic area (oncology)	New use in similar indication considered as repositioning	(1)
Travoprost	Reduction and subsequent control of intraocular pressure (IOP) in primary open angle glaucoma (POAG)	–	Benzalkonium chloride (BAC) as preservative system	BAC-free formulation	Ref.	Reformulation by change of preservative system	Excipient change considered as reformulation	(32)
Warfarin	Thrombosis prevention	Secondary prophylaxis following myocardial infarction	–	–	Rep.	New indication, Similar therapeutic area	New use in similar indication considered as repositioning	(31)

A list of all cases found in the literature search for which incoherent classification was attributed is presented in this table. Details such as original indication and formulation were provided along with the new indication and/or formulation and the classification as attributed in the literature by their respective authors.

a Ref.: Reformulation; Rep.: Repositioning; Ref.-Rep.: Reformulation mid Repositioning.

One of the common points in the different definitions used by different authors was that drug repositioning is defined as the process of finding a new use for an already known drug. However, definitions diverged in several parameters, such as the exact meaning of ‘new use’ and ‘known drug’. Some authors consider a new indication within the same/similar therapeutic use as repositioning, while others exclude this category from the repositioning definition. Another case was the so-called ‘geographic repositioning’, which is considered by some authors as a repositioning category while this particular case does not meet the definition criteria of discovery of a new use.

Some authors considered reformulation as part of drug repositioning efforts. Also, differences regarding the classification of types of reformulation were found in the literature.

### Proposal of a harmonized nomenclature

The previously described absence of a common, clear, and concise definition for repositioning and reformulation of drugs prompted us to propose a global definition that attempts to cover all of the previously unclear and sometimes contradictory criteria for the definitions and classification of cases of repositioning and reformulation.

- **Drug repurposing**
  The term repurposing includes all the re-development strategies based on the same chemical structure of the therapeutically active ingredient as in the original product. More precisely, under this term, we regroup drug repositioning, reformulation, and combination strategies.
- **Drug repositioning**
  Repositioning is the process of finding a new indication for a drug or compound. By this, we assume that the new indication is distinct from the already approved/intended indication of the original product, where ‘distinct’ implies an anatomical and/or therapeutically distinct indication referring to the 10th version of the International Classification of Diseases (ICD-10). The situation where the new indication involves a different pharmacological target (off-target repositioning) is the only exception where a new use in a similar indication will be covered by the actual definition.
  The original product candidate to drug repositioning should fall into one of the following categories:
- **Drugs that were never commercialized**
  Drugs in clinical development
  Drugs with finalized but failed/negative clinical development due to poor efficacy profile and/or less frequency due to efficacy issues
  Drugs that were not completely developed, particularly from academic institutions and public sector laboratories
- **Drugs that are/were commercialized**
  Drugs that have been marketed but discontinued for commercial reasons
  Drugs that have been marketed but discontinued for safety/public health reasons
  Marketed drugs for which IP rights are still in place
  Marketed drugs for which patents are already expired or when generic versions are already available in the market.
  Some authors also consider drugs that have been discovered, developed, and marketed in small or emerging markets but not widely launched nor launched in larger pharmaceutical markets, especially in the United States and Europe, as potential candidates for drug repositioning (1, 7, 33). Those authors consider this phenomenon as ‘geographic’ or ‘transnational’ repositioning of drugs. However, referring to our basic definition of repositioning of drugs, we found that this category should be excluded for consistency.
- **Reformulations**
  Reformulation is, by the simple definition of the term, making a particular change in the formulation of the original drug. This can be achieved by exploiting advances in formulation technology to change the release of the active substance, pharmaceutical forms, and/or route of administration but it can also concern some excipients with no impact on the pharmacokinetic parameters. No change should be incurred in the structure of the API except when it is a chiral switch. By this, we assume that approaches modifying the chemical structure of the API, such as prodrugs or active metabolites, are not included in our definition. Cases where the development of a new product does not include a change in the original formulation (i.e., change of dose, package size, etc.) should also be excluded.
- **Repositioning aided by reformulation**
  Repositioning aided by reformulation is a particular situation wherein a new formulation (new administration route, new pharmaceutical form, among others) is developed for a new therapeutic use of a candidate for repositioning. To be considered as a repositioned and reformulated product, the change incurred to the original product should adhere to both aforementioned definitions.
- **Drug combination**
  Regarding the specific case of drug combinations, we concluded that drug combinations can be considered a separate strategy, and where criteria of definitions are met, they can fall under the classification of reformulation and/or repositioning. By this, combinations can be classified as cases of reformulation or repositioning provided there was a change in formulation or indication, respectively.

### Proposal of taxonomy for drug repositioning and reformulation (algorithm)

**Fig. 3:**
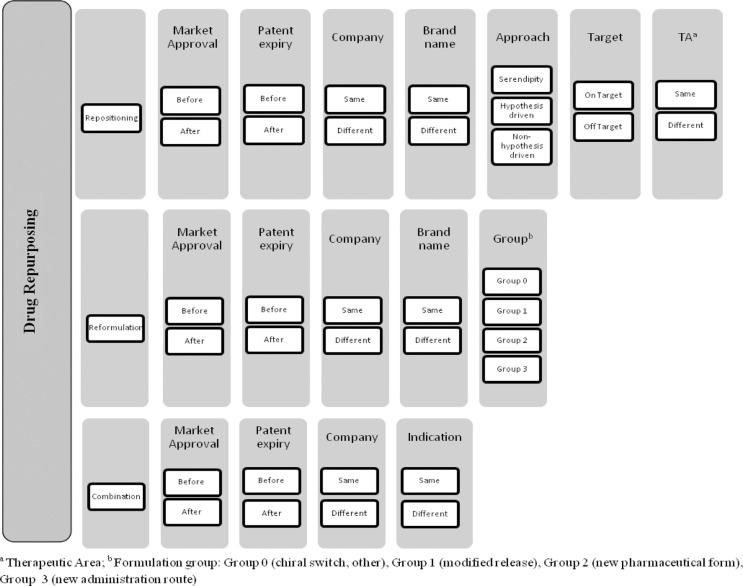
Classification algorithm for drug repurposing. Drug repurposing strategies (i.e., repositioning, reformulations, and combination) are illustrated as three distinct lines. Under each of these strategies, the cases can be classified according to specific criteria.

Based on the proposed definitions and from our understanding of actual classifications, we propose a complete classification algorithm (Fig. 3) for drug repurposing.

The chosen parameters for classification reflect our evaluation of the attributes having a direct or indirect impact on the development of products, particularly on their market access conditions. The combination of factors such as the commercialization and genericization status of the original product at the time of launching the new product, being developed by the same or a different company, under the same or a different brand name as for the original product, leads to a large set of development scenarios.

**Fig. 4:**
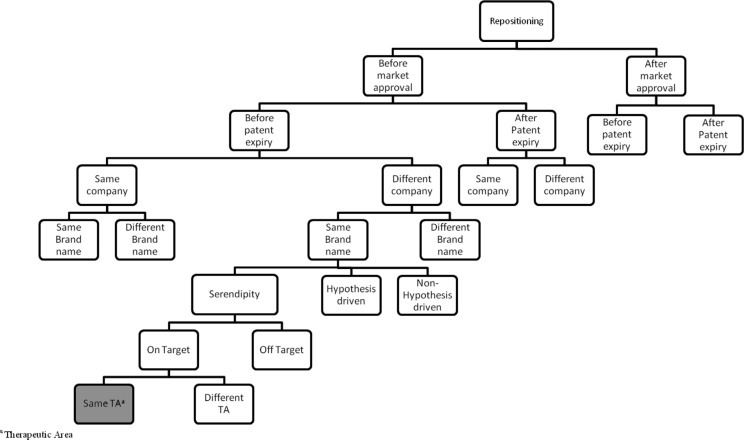
Classification tree for repositioning of drugs. Cases of drug repositioning are classified according to the parameters mentioned in the algorithm (Fig. 3). The presented tree is only a partial illustration of the classification.

More specifically, cases of repositioning (Figs. 3 and 4) are classified referring to the approach leading to the discovery of the new indication (approach). We opted for three possible approaches in our classification for repositioning of drugs: serendipity, hypothesis-driven- and non-hypothesis-driven-strategies (see definitions in Supplemental material 2). Moreover, cases of drug repositioning can be classified via two other parameters closely related to the nature of the indication switch, that is, pharmacological target and therapeutic area compared to the original product. It should be noted that cases where the new indication is within the same therapeutic area via the same pharmacological target will be excluded.

**Fig. 5:**
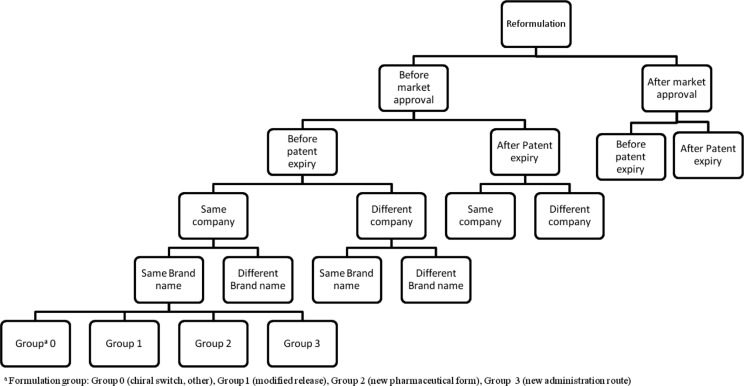
Classification tree for drug reformulation. Cases of drug reformulation are classified according to the parameters mentioned in the algorithm (Fig. 3). The presented tree is only a partial illustration of the classification.

Concerning drug reformulation (Figs. 3 and 5), in addition to the common classification criteria, we opted for a four-group classification, regarding the type of formulation change, where groups 0–3 were defined as follows:

Group 0: Chiral switch, excipient change without pharmacokinetic impact and cases where none of the other classification of groups (1, 2, or 3) is applicable;

Group 1: Same pharmaceutical form, same administration route, and different pharmacokinetic parameters (e.g., modified release formulations);

Group 2: Different pharmaceutical form, same or similar administration route, and same pharmacokinetic parameters;

Group 3: Different pharmaceutical form and different administration route.

**Fig. 6:**
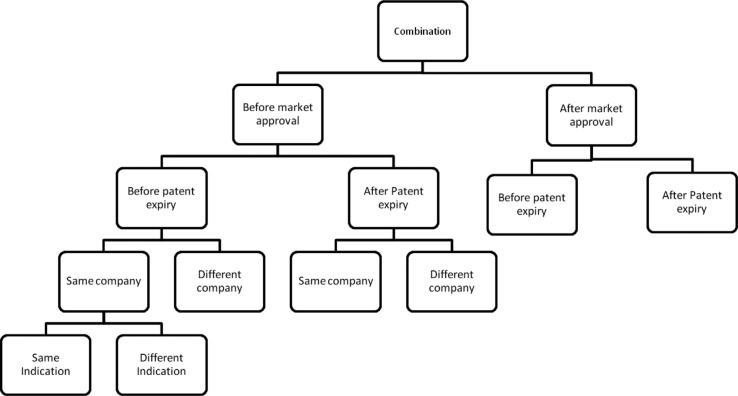
Classification tree for drug combination. Cases of drug combination are classified according to the parameters mentioned in the algorithm (Fig. 3). The presented tree is only a partial illustration of the classification.

Finally, the classification of drug combinations (Figs. 3 and 6) is dependent on whether the new mixture is indicated for the same or a different therapeutic use compared with the indications already granted for the individual components.

### Evaluation and possible limits of the proposed nomenclature and taxonomy

Although our proposal aims to be universal yet precise in its content, some cases may be beyond its purview. Due to the ultimate goal of analyzing the association of pricing and reimbursement regulations on development of repurposed drugs, we have excluded in-development or non-approved cases and cases for which data could not be retrieved. As such, we cannot extrapolate the utilization of our proposed nomenclature to all possible existing cases of repositioning or reformulation. For all new cases not included due to date limitation inherent to the current literature review, we suggest that these can be analyzed and categorized for their nomenclature as soon as they are known and eventually the proposed nomenclature be re-evaluated in light of these future findings, if necessary.

## Conclusion

Given the disparity and inconsistency of terminologies and classifications found in the literature, a harmonized nomenclature for repositioning, reformulation, and combinations will allow a robust and consistent analysis of the added value and market access conditions attributed for each strategy and case type as assessed by regulators and payors in Europe and the United States.

## Supplementary Material

Drug reformulations and repositioning in pharmaceutical industry and its impact on market access: reassessment of nomenclature

Drug reformulations and repositioning in pharmaceutical industry and its impact on market access: reassessment of nomenclature
