# Supplementary figures and images for: Drug reformulations and repositioning in pharmaceutical industry and its impact on market access: reassessment of nomenclature
Source: J Mark Access Health Policy. 2013 Aug 6;1:10.3402/jmahp.v1i0.21131. doi: 10.3402/jmahp.v1i0.21131 (PMC4865745; doi:10.3402/jmahp.v1i0.21131)

## Slide 1
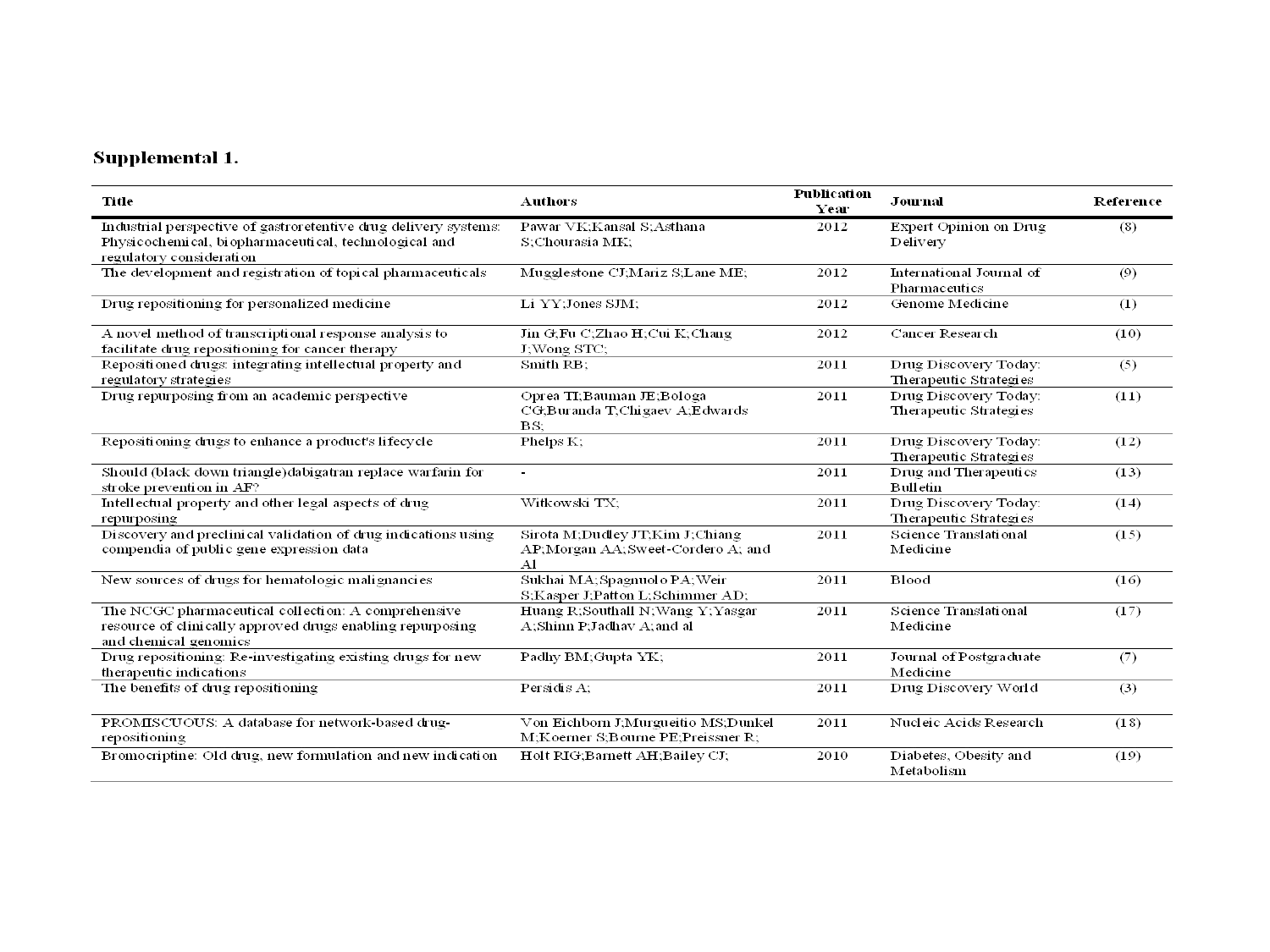

## Slide 2
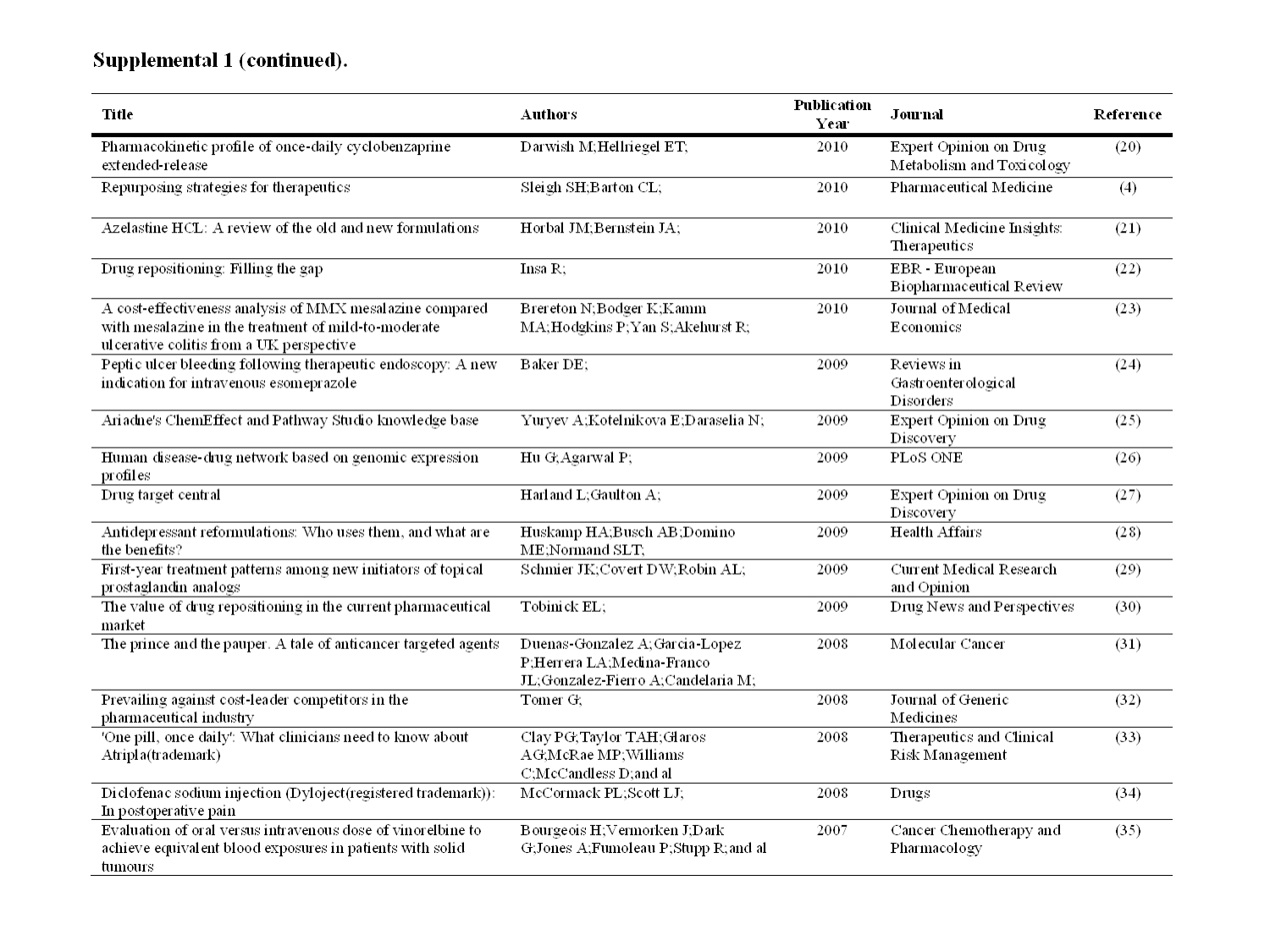

## Slide 3
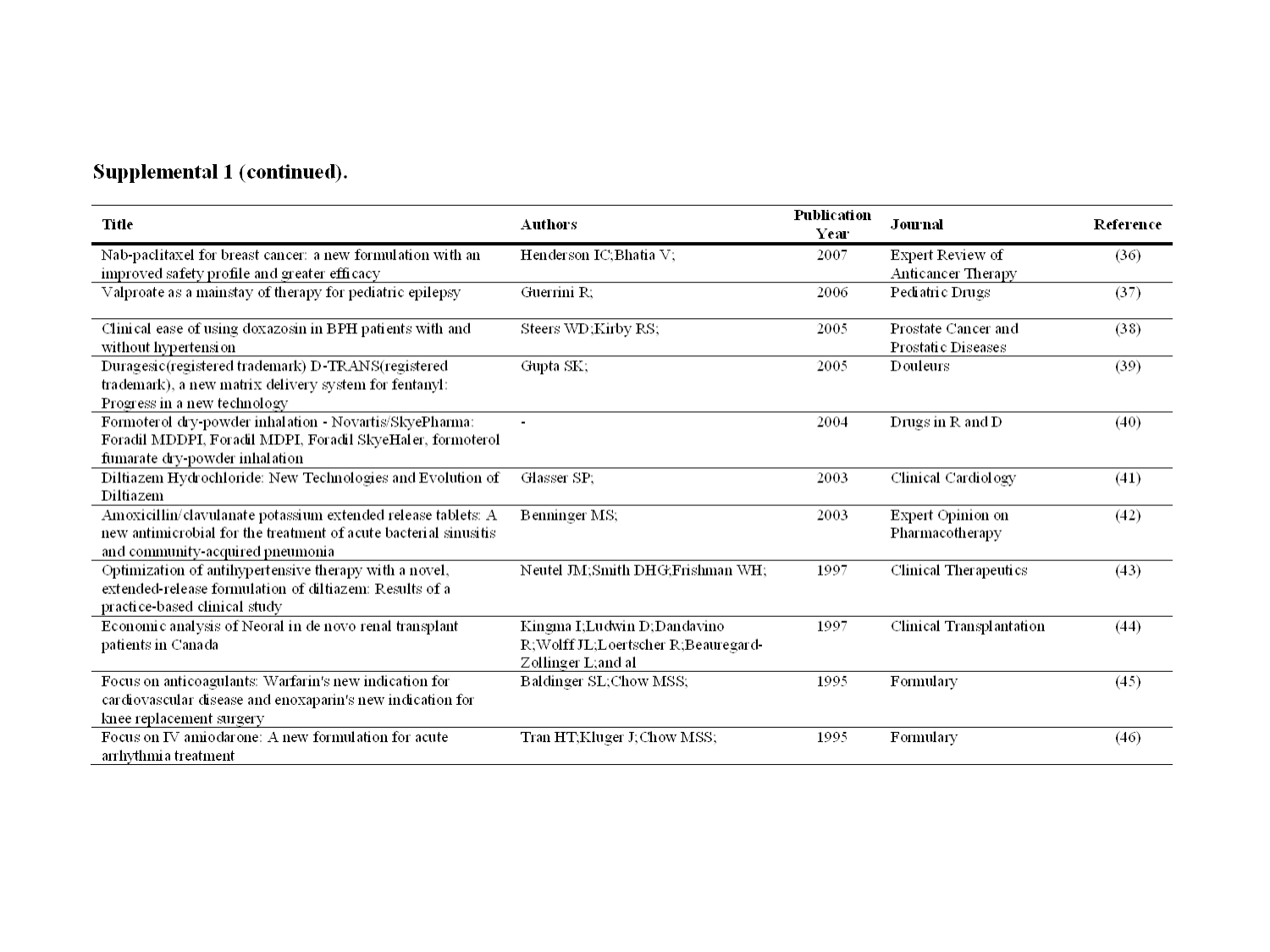

Supplement: Drug reformulations and repositioning in pharmaceutical industry and its impact on market access: reassessment of nomenclature [file JMAHP-1-21131-s001.pptx]
