# Supplementary material for: Drug reformulations and repositioning in pharmaceutical industry and its impact on market access: reassessment of nomenclature
Source: J Mark Access Health Policy. 2013 Aug 6;1:10.3402/jmahp.v1i0.21131. doi: 10.3402/jmahp.v1i0.21131 (PMC4865745; doi:10.3402/jmahp.v1i0.21131)
Supplement: Drug reformulations and repositioning in pharmaceutical industry and its impact on market access: reassessment of nomenclature [file JMAHP-1-21131-s002.docx]

**Supplemental 2. Repositioning approaches**

Historically, new indications for existing drugs were discovered serendipitously. Famous examples of serendipity repositioning are sildenafil, minoxidil and thalidomide. However, during the past decade a shift from serendipitous identification of alternative therapeutic use of a product towards a more systematic exploitation of the relationships between disease, proteins and small molecules has been observed.(27) Additionally to fortuitous observations, ideas for repositioning can come from informed insights theory or from novel research including *in vivo* assays, *in vitro/ ex vivo* assays and computational approaches (*in silico*).

The development of repositioning products can be conducted and achieved through different processes and techniques. The main strategies found through the review of the literature and proposed classification for the discovery and development proposed by different authors are enumerated bellow:

- **Serendipity:** pertains to the fortuitous discovery of a new clinical potential for the molecule made while looking for something unrelated. This can occur through the clinical observation during the clinical use for its original purpose or the interpretation of adverse events observed during clinical trials for the original indication.(16;22) Serendipity has often been instrumental in prompting investigators to test a drug for a new indication (47) and led so far to the identification the most commercially successful repositioned cases.(1)
- **Hypothesis driven (Informed insight):** In contrast to the serendipitous indication discovery, a more rational approach -depending less on chance- is being increasingly implemented by drug repositioning players and particularly specialist companies.(4) Hypothesis driven repositioning is also labelled informed insight or the rational scientific approach. It relies on an in-depth understanding of the disease biology and connecting what is currently known about a drug to estimate possible novel uses.(16;47) For previously commercialized products it is sometimes considered a sophisticated form of drug lifecycle management (LCM) strategy by some authors.(50) Because it is a directed approach and based on known facts, some authors argue that it will never predict unknown or non-obvious activities thus not necessarily leading to the exploration of the molecules’ full therapeutic potential.(16;47)
- **Novel platforms (Non hypothesis driven**): Technology platforms can be used to develop a systematic approach to drug repositioning.(4) Since these platforms allow large screening, they are usually looking for unknown or unexpected results via the concept of ‘fishing’.(22) A range of technologies are applied for repositioning and some authors (1) divide those strategies into two categories: the experimental approaches (*in vitro* and *in vivo*/ *ex vivo* assays) and the computational methods.

**Experimental approaches (in vivo/in vitro/ex vivo)**

- *In vivo* assays:

*Non-invasive imaging*: uses real-time in vivo imaging capabilities, which allow the development of spatiotemporal pharmacodynamic profiles for a drug. This can reveal previously unknown sites of action.(50)

*Animal models:* screening for the amelioration of a disease phenotype in an animal model (51)

- *In vitro / ex vivo* assays:

*Binding assays:* are used to determine potential direct binding partners or ligands for existing drugs. Examples of this approach include washing cell lysate extracts over a bead column fixed with an approved drug, or high-throughput direct-binding assays to test drugs against 317 kinases (1),

*Cell-based approaches:* screen used for drugs that induce a desired change in cellular phenotype. These approaches have been used to find approved drugs that can regulate autophagy, induce apoptosis in retinoblastoma cell lines, or inhibit proliferation of prostate cancer cell lines.(1)

*Genome expression:* this technique is used to identify drugs that have an opposite gene expression profile to that of a disease or having similar gene expression profiles in cell lines to other approved drugs.(1)

There are some studies that have combined some of the previous described processes to retrieve a repositioning product such as combined use of high-throughput cell proliferation, kinome binding assays and in vivo mouse studies.(1) Additionally, other technologies were mentioned by some authors among which we cite the recepterome platforms, and flow cytometry.(11;22)

**Computational approaches (*in silico*; Data mining; Gene expression and High throughput screening)**

In silico approaches make use of recent bioinformatics advances (genomics, proteomics, structural, functional (7)) to identify potential drug repositioning opportunities.(16) These approaches often combine knowledge of the drug target space with pharmacological data and literature analysis.(18)

*Molecular docking and inverse docking*: is a high resolution method which simulates the binding of a drug inside a three-dimensional structure target at an atomic level. This approach is used to virtually screen large chemical libraries against targets of interest. In contrast, inverse docking was at first proposed to investigate the docking of one drug against multiple protein binding sites, but then methods were scaled up to allow the investigation of hundreds of targets and thousands of drugs.(1)

*Data mining*

The search for promising compounds often involves a review of the public and subscription-based information sources such as company websites, intellectual property (IP) and scientific databases.(7) Examples of network analysis are MANTRA (a computational tool for the analysis of the Mode of Action (MoA) IDMap (a java-based software that enables researchers to map commercial chemicals to possible drug targets), or CoPub (a text mining tool that detects co-occuring biomedical concepts in abstracts from the MedLine literature database (52,53)).(1)

*Systems biology*

New target-disease associations can also be formed using systems biology approaches (1)) which attempt to mathematically model the complex physiological conditions of health and disease as an interdependent signaling network of biological elements and pathways.(47)

*Gene expression*

Certain specialized companies rely on microarray-based, target- and mechanism-independent drug discovery platforms. Verva Pharmaceuticals for example has developed the Gene Expression Signature (GES) platform, which is built on technology that was originally developed for cancer research.(47)

Other computational approaches were mentioned in the literature including high throughput screening (HTS) and databases of clinical side effects. No single current technology is powerful enough to completely eliminate the risk of failure associated with conduction of clinical trials in patients. However, in order to confirm and increase the confidence in the development potential of a repositioned drug as much as possible, a portfolio of complementary technologies should be assembled to provide independent confirmation of the original technology’s findings.(22)
